# Thickness dependence of anomalous Nernst coefficient and longitudinal spin Seebeck effect in ferromagnetic Ni_x_Fe_100−x_ films

**DOI:** 10.1038/s41598-017-05946-1

**Published:** 2017-07-21

**Authors:** Harsha Kannan, Xin Fan, Halise Celik, Xiufeng Han, John Q. Xiao

**Affiliations:** 1 Department of Physics and Astronomy, University of Delaware, Newark, DE 19716 USA; 2 State Key Laboratory of Magnetism, Institute of Physics, Chinese Academy of Science, Beijing, China

## Abstract

Spin Seebeck effect (SSE) measured for metallic ferromagnetic thin films in commonly used longitudinal configuration contains the contribution from anomalous Nernst effect (ANE). The ANE is considered to arise from the bulk of the ferromagnet (FM) and the proximity-induced FM boundary layer. We fabricate a FM alloy with zero Nernst coefficient to mitigate the ANE contamination of SSE and insert a thin layer of Cu to separate the heavy metal (HM) from the FM to avoid the proximity contribution. These modifications to the experiment should permit complete isolation of SSE from ANE in the longitudinal configuration. However, further thickness dependence studies and careful analysis of the results revealed, ANE contribution of the isolated FM alloy is twofold, surface and bulk. Both surface and bulk contributions, whose magnitudes are comparable to that of the SSE, can be modified by the neighboring layer. Hence surface contribution to the ANE in FM metals is an important effect that needs to be considered.

## Introduction

Spintronics which deploys the spin, in addition to or sometimes in place of the charge of the electron has exhibited rich physics and industrial potential in the past two decades. Intensive research has been carried out investigating the interaction among, spin and magnetic field^1^, electric current^2–4^, electromagnetic waves^5^ and recently temperature gradient^6^. A temperature gradient in a ferromagnetic metal generates a spin chemical potential splitting between spin up and spin down electrons, owing to the spin dependent density of states^7^. This leads to a spin current with no net charge flow in an open circuit, and is called the Spin Seebeck effect. First reported in ferromagnetic metals^7^, SSE was later found to be present in ferromagnetic semiconductors^8^ and insulators^9, 10^. The inverse Spin Hall Effect (ISHE)^11^ is a common tool employed to measure SSE, where a HM, for example Pt, placed adjacent to the ferromagnet converts the spin current into a charge current.

**Figure 1:**
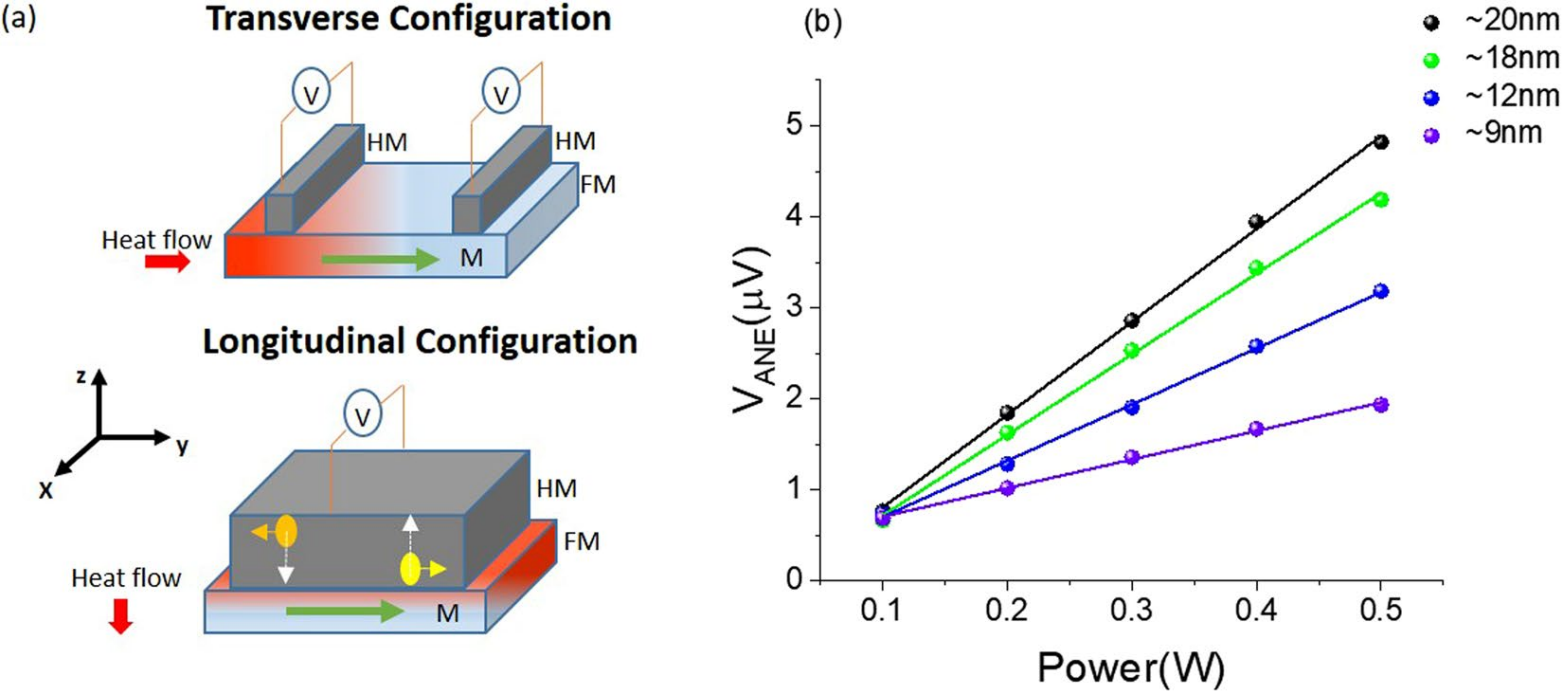
(**a**) Illustration of transverse and longitudinal configurations for detecting the spin Seebeck effect. (**b**) Power dependence of measured anomalous Nernst signal for different thicknesses of Ni_7_Fe_93_ sample.

Thermal measurements are typically carried out in the longitudinal and transverse geometries^12^. As illustrated in Fig. 1(a), in the longitudinal configuration, the temperature gradient is applied normal to the plane of the ferromagnet whereas in the transverse configuration, the temperature gradient is applied in the plane of the ferromagnet. However it has been demonstrated that the SSE signal measured in the transverse configuration may be contaminated with an out-of-plane temperature gradient due to the finite thermal conductivity of the substrate^13^. The longitudinal configuration has a well-defined direction for the temperature gradient and is suitable for the propagation of spin current due to the short spin-diffusion length.

There are several obstacles in implementing the longitudinal configuration to the SSE. Signal from ANE^14^ of the ferromagnet favors the same geometry as the SSE. Due to magnetic proximity effect^15^, a heavy metal (HM) in contact with a FM may produce an anomalous Nernst signal that has the same profile as the SSE. Hence complete isolation of SSE in FM metals has been a profound challenge. In this report, we discuss the fabrication of a metallic FM alloy with “zero anomalous Nernst coefficient”, which helps eliminate the ANE signal generated by the FM, and perform longitudinal SSE (LSSE) measurements using the alloy while separating HM from FM with a thin layer of a non-magnetic (NM) metal, Cu to avoid the proximity contribution. Such modifications to the LSSE experiment in principle should allow the isolation of pure SSE from other parasitic effects. However, thickness dependence studies of the zero anomalous Nernst alloy revealed, in addition to the bulk contribution, the existence of a significant surface contribution to the ANE. Such a surface contribution will be modified with different neighboring NM layers, particularly in the case of a heavy metal.

In a simplified scenario, assuming a transparent interface, the spin diffusion in the FM/HM bilayer, in the longitudinal configuration can be described by the one-dimensional drift-diffusion model^6, 16^,

1 $$\{\begin{array}{c}{{\rm{\nabla }}}^{2}({\mu }_{\uparrow }-{\mu }_{\downarrow })=\frac{1}{{\lambda }^{2}}({\mu }_{\uparrow }-{\mu }_{\downarrow })\\ {j}_{c}=-{\rm{\nabla }}({\sigma }_{\uparrow }{\mu }_{\uparrow }+{\sigma }_{\downarrow }{\mu }_{\downarrow })+({\sigma }_{\uparrow }{S}_{\uparrow }+{\sigma }_{\downarrow }{S}_{\downarrow })(-{\rm{\nabla }}T)\\ {j}_{s}=-{\rm{\nabla }}({\sigma }_{\uparrow }{\mu }_{\uparrow }-{\sigma }_{\downarrow }{\mu }_{\downarrow })+({\sigma }_{\uparrow }{S}_{\uparrow }-{\sigma }_{\downarrow }{S}_{\downarrow })(-{\rm{\nabla }}T)\end{array}$$

where *j*_*c*_ the charge current density, *j*_*s*_ the spin current density, *λ* the spin diffusion length, and $\nabla T$ the temperature gradient. *μ*, *σ*, and *S* are spin electrochemical potential, electrical conductivity and Seebeck coefficient respectively. Here the subscripts ↑ and ↓ denote spin-up and spin-down electrons, respectively. Spin current distribution obtained by applying boundary conditions, that the charge current vanishes everywhere and the spin current vanishes at the two surfaces while being continuous at the FM/HM interface is,

2 $$\{\begin{array}{c}{j}_{s{\rm{\_}}FM}(z)={\sigma }_{FM}({S}_{\uparrow }-{S}_{\downarrow }){\rm{\nabla }}T[1-{\textstyle \tfrac{\begin{array}{c}{\textstyle \tfrac{{\lambda }_{HM}}{{\lambda }_{FM}}}{\textstyle \tfrac{{\sigma }_{FM}}{{\sigma }_{HM}}}\,{\rm{C}}{\rm{o}}{\rm{s}}{\rm{h}}\,({\textstyle \tfrac{{d}_{HM}}{{\lambda }_{HM}}})[{\rm{S}}{\rm{i}}{\rm{n}}{\rm{h}}\,({\textstyle \tfrac{{d}_{FM}+z}{{\lambda }_{FM}}})-\,{\rm{S}}{\rm{i}}{\rm{n}}{\rm{h}}\,({\textstyle \tfrac{z}{{\lambda }_{FM}}})]+\,{\rm{C}}{\rm{o}}{\rm{s}}{\rm{h}}\,({\textstyle \tfrac{z}{{\lambda }_{FM}}})\,{\rm{S}}{\rm{i}}{\rm{n}}{\rm{h}}\,({\textstyle \tfrac{{d}_{HM}}{{\lambda }_{HM}}})\end{array}}{[{\textstyle \tfrac{{\lambda }_{HM}}{{\lambda }_{FM}}}{\textstyle \tfrac{{\sigma }_{FM}}{{\sigma }_{HM}}}\,{\rm{S}}{\rm{i}}{\rm{n}}{\rm{h}}\,({\textstyle \tfrac{{d}_{FM}}{{\lambda }_{FM}}})\,{\rm{C}}{\rm{o}}{\rm{s}}{\rm{h}}\,({\textstyle \tfrac{{d}_{FM}}{{\lambda }_{HM}}})+\,{\rm{C}}{\rm{o}}{\rm{s}}{\rm{h}}\,({\textstyle \tfrac{{d}_{FM}}{{\lambda }_{FM}}})\,{\rm{S}}{\rm{i}}{\rm{n}}{\rm{h}}\,({\textstyle \tfrac{{d}_{HM}}{{\lambda }_{HM}}})]}}]\\ {j}_{s{\rm{\_}}HM}(z)={\sigma }_{FM}({S}_{\uparrow }-{S}_{\downarrow }){\rm{\nabla }}T[{\textstyle \tfrac{{\rm{S}}{\rm{i}}{\rm{n}}{\rm{h}}\,({\textstyle \tfrac{{d}_{HM}-z}{{\lambda }_{HM}}})[{\rm{C}}{\rm{o}}{\rm{s}}{\rm{h}}\,({\textstyle \tfrac{{d}_{FM}}{{\lambda }_{FM}}})-1]}{[{\textstyle \tfrac{{\lambda }_{HM}}{{\lambda }_{FM}}}{\textstyle \tfrac{{\sigma }_{FM}}{{\sigma }_{HM}}}\,{\rm{S}}{\rm{i}}{\rm{n}}{\rm{h}}\,({\textstyle \tfrac{{d}_{FM}}{{\lambda }_{FM}}})\,{\rm{C}}{\rm{o}}{\rm{s}}{\rm{h}}\,({\textstyle \tfrac{{d}_{HM}}{{\lambda }_{HM}}})+\,{\rm{C}}{\rm{o}}{\rm{s}}{\rm{h}}\,({\textstyle \tfrac{{d}_{FM}}{{\lambda }_{FM}}})\,{\rm{S}}{\rm{i}}{\rm{n}}{\rm{h}}\,({\textstyle \tfrac{{d}_{HM}}{{\lambda }_{HM}}})]}}]\end{array}$$

where *d* is the thickness, $\sigma =\frac{2{\sigma }_{\uparrow }{\sigma }_{\downarrow }}{{\sigma }_{\uparrow }+{\sigma }_{\downarrow }}$ and the subscripts FM and HM denotes those for the ferromagnet and heavy metal respectively. The spin current in the HM measured through ISHE can be used to determine the strength of the SSE in the FM. It can be understood from Eq. (2), that more spin current goes into the HM when its spin diffusion length is shorter and conductivity is higher than those of the FM^17–20^. Therefore, Pt is the optimum candidate as the HM for the longitudinal SSE measurement.

## Methods and Results

**Figure 2:**
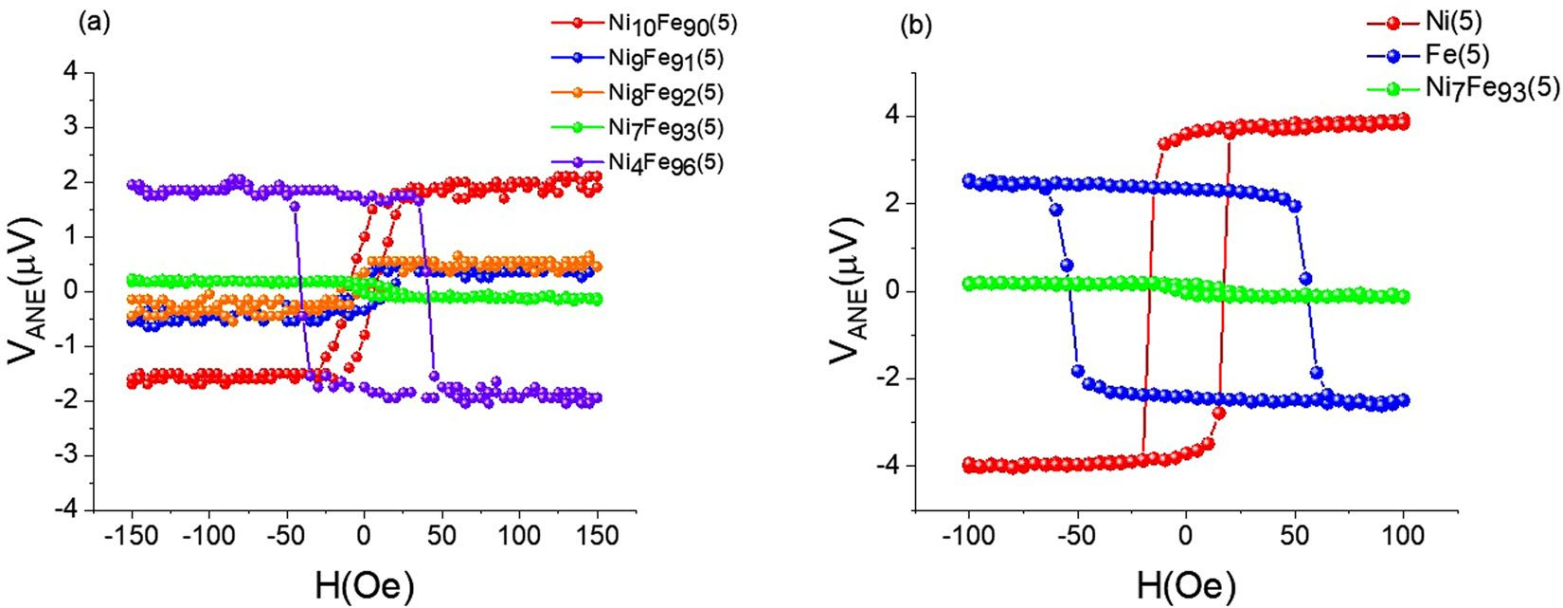
(**a**) Anomalous Nernst effect measurement for select Ni_x_Fe_100−x_(5) compositions. (**b**) Comparison of anomalous Nernst signal of Ni(5), Fe(5) and, Ni_7_Fe_93_(5).

The use of a FM with zero Nernst coefficient will be an ideal experimental solution to separate SSE from ANE of the FM. To fabricate a FM with zero anomalous Nernst coefficient, two typical FM metals, Fe and Ni, known to have opposite anomalous Nernst coefficients^21, 22^, can be alloyed. Subsequent to target preparation, all necessary thin films were fabricated on a wet thermally oxidized Silicon wafer with magnetron sputtering at a base pressure lower than 5 × 10^−7^ torr, 4.5 mtorr working pressure and 10 sccm Argon flow. All samples were capped with a 5 nm SiO_2_ layer. Which serves two purposes, it prevents samples from oxidation and electrically insulates heater from the sample. Fabricated samples were defined into 15 mm × 2 mm pieces and pasted on to a thick Cu block using thermal grease. Heater is placed on top and maintained at fixed power. As shown in Fig. 1(b), the measured ANE signal is linear with the heater power. Figure 2(a) shows the anomalous Nernst measurements for select Ni_x_Fe_100−x_(5) (number in the parentheses is the thicknesses in nanometers) nominal compositions. By tuning the composition of the alloy Ni_x_Fe_100−x_, where we invoke the competition between opposite anomalous Nernst signals of constituent elements, achieved zero Nernst coefficient in Ni_7_Fe_93_ at a thickness of 5 *nm*. Figure 2(b) compares the anomalous Nernst signal of optimized composition Ni_7_Fe_93_(5) with that of pure Ni(5) and Fe(5). Optimized composition has a profile similar to pure Fe and an anomalous Nernst coefficient at least 20 times smaller than that of Ni or Fe, and hence considered here as the “zero Nernst material”.

**Figure 3:**
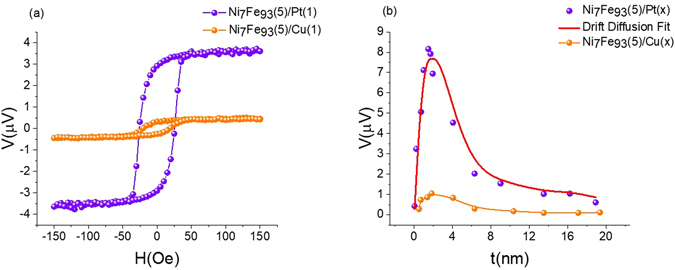
(**a**) The thermal voltage measured for Ni_7_Fe_93_(5)/Pt(1) and control sample Ni_7_Fe_93_(5)/Cu(1). Films are heated under the same heater power as in Fig. 2. Signal for sample with Pt is more than 20 times larger than that for Cu. (**b**) The thickness dependence of the thermal voltage. Curve for Ni_7_Fe_93_(5)/Pt(x) peaked when Pt thickness is about 2 nm. The parameters for the fitting are:${\lambda }_{Pt}=1.3\,nm$, ${\lambda }_{FM}=5\,nm$, ${d}_{FM}=5\,nm$, ${\sigma }_{Pt}=5\,a.u.$, ${\sigma }_{FM}=1\,a.u.$ *and*, $({S}_{\uparrow }-{S}_{\downarrow })\nabla T=1\,a.u.$ Curve for Ni_7_Fe_93_(5)/Cu(x) does not show any significant variation with the thickness of Cu except a little bump around 2 nm.

In Fig. 3(a), a representative curve of Ni_7_Fe_93_(5)/Pt(1) is shown. When a HM, Pt is placed adjacent to Ni_7_Fe_93_(5), the “zero Nernst material”, a Nernst-like voltage can be observed due to the ISHE in Pt. The measured Nernst-like voltage is in the same polarity as that of a pure Ni, discussed above. Recall signal for Ni_7_Fe_93_(5) was in the same polarity as pure Fe. As a comparison, the curve for a control sample Ni_7_Fe_93_(5)/Cu(1) is shown in the same figure with a signal 20 times smaller. An electric field arises in Pt due to the ISHE, ${E}_{ISHE}={\rho }_{Pt}{\theta }_{SH}{j}_{s\_HM}$ where *ρ*_*Pt*_ is the resistivity of the Pt layer, *θ*_*SH*_ is the spin Hall angle. Considering the shorting of the adjacent FM layer, the measured voltage can be derived as Eq. (3), where the length of the sample under temperature gradient is *L* and, *R* is the sheet resistance of the sample.

3 $${V}_{ISHE}=R{\theta }_{SH}L{\int }_{0}^{{d}_{Pt}}{j}_{s}(z)dz$$

Thickness dependence study of Pt and control sample with Cu are shown in Fig. 3(b). Since the spin diffusion length is very short in Pt, the spin current distribution in Ni_7_Fe_93_(5)/Pt(x) bilayer will remain unchanged as Pt gets thicker. Thus, measured voltage scales with the total resistance. Indeed, as shown in Fig. 3(b), the SSE voltage increases quickly with the Pt thickness up to about 2 *nm*, followed by monotonic decay as the Pt thickness increase further. The red curve is a fitting based on Eq. (3), which gives rise to a spin diffusion length in Pt to be about 1.3 *nm*. This result is consistent with the values extracted from the spin pumping measurements^23^. Signal for the control sample Ni_7_Fe_93_(5)/Cu(x) does not show any significant variation except a little bump around 2 *nm*.

**Figure 4:**
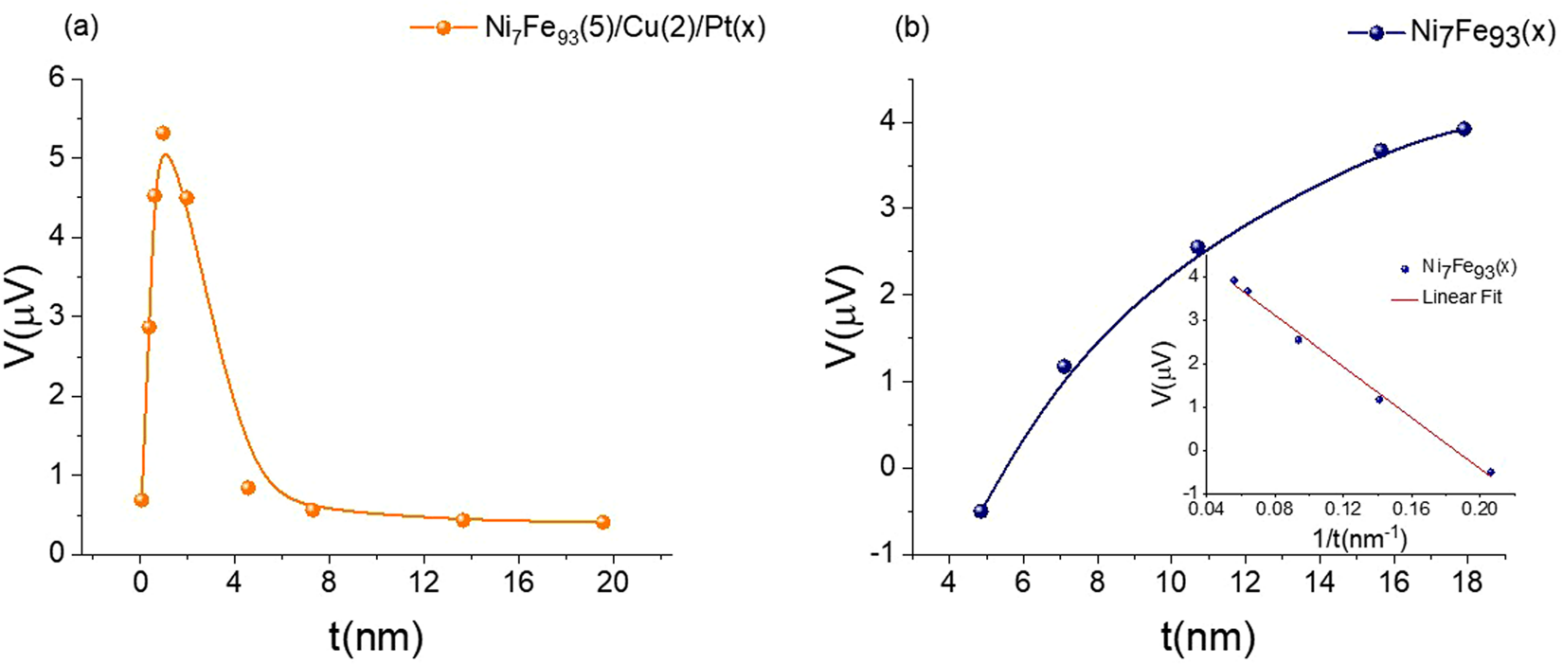
(**a**) Thermal voltage measured for Ni_7_Fe_93_(5)/Cu(2)/Pt(x). Thickness dependence is similar to the case without Cu insertion, except for reduction in magnitude, owing to shorting due to Cu and change in the transparency for spin current with the insertion of the Cu layer. (**b**) Thermal voltage measured for Ni_7_Fe_93_(x) and the inset shows the thermal voltage dependence on inverse thickness.

An interface effect such as proximity effect^15^ can be surfaced as a possible explanation for the signal measured for Ni_7_Fe_93_(5)/Pt(x). When Pt is in contact with a FM, it may be partially magnetized giving rise to anomalous Nernst signal that has the same profile as the SSE. Due to the short spin diffusion length, it is difficult to distinguish the SSE and the proximity effect from the length scale fitting in Fig. 3(b). Insertion of a thin piece of Cu in between Ni_7_Fe_93_ and Pt help eliminate the proximity effect while preserving the SSE, since the spin current can penetrate the Cu layer. Cu is a typical diamagnetic metal, far from Stoner instability^24, 25^, hence will not give rise to any SSE signal. Moreover, since spin Hall angle of Cu is negligibly small^26^ it does not produce any appreciable inverse spin Hall voltage as well. Based on these facts, Cu is an ideal choice for the insertion layer between the FM and the HM. As illustrated in Fig. 4(a), Ni_7_Fe_93_(5)/Cu(2)/Pt(x) sample still shows a sizable Nernst-like voltage. The magnitude is reduced due to the shunting effect, that the Cu shorts the voltage. Another possible reason for the reduction of the magnitude is the change of transparency to spin current at the interface so that the spin current entering Pt is reduced. Despite the reduction of the magnitude, comparing Figs 3(b) and 4(a) one can still observe the same trend in thickness dependence with and without the insertion of Cu. Stack of thin films along with the substrate shall be treated as series thermal resistors for heat conduction purposes. Substrate thickness, *t*_*sub*_ (=500 *μm*) is much larger compared to *nm* scale thicknesses of the thin films and the thermal conductivity of the substrate, *k*_*sub*_ ($\ll$ *k*_*Pt*_, *k*_*Cu*_, *k*_*NiFe*_) is much smaller than thermal conductivities of the thin films. Hence the thermal resistance, *R*_*sub*_ (∝$\tfrac{t}{k}$) of the substrate completely overwhelms the heat conduction process. Therefore, within the thickness range of the top layer we probed, variation in the temperature gradient can be considered negligibly small.

By adopting Ni_7_Fe_93_(5), a “zero Nernst material” as the FM and by inserting a thin layer of Cu between Ni_7_Fe_93_(5) and Pt, we have mitigated, two major contributions, ANE and proximity effect, eclipsing the LSSE measurements. To further solidify the results, a thickness dependence study has been performed on the Ni_7_Fe_93_, the FM metal used in the experiment. The results are shown in Fig. 4(b). Inset of Fig. 4(b) shows the inverse thickness dependence. This result is very interesting and merits detailed discussion.

## Discussion

The anomalous Nernst voltage measured for Ni_7_Fe_93_, varies with the thickness, suggesting the bulk anomalous Nernst contribution is not absolute, whereas a linear inverse thickness dependence suggests the existence of a surface anomalous Nernst contribution. The inverse thickness dependence shown in the inset of Fig. 4(b) is fitted with a linear curve by Eq. (4). It should be noted, though the resistivity of the Ni_7_Fe_93_ depends on the film thickness, behavior of the ANE signal does not directly correspond to the resistivity, a sign change in the ANE signal can no way be explained by the resistivity.

4 $$V={V}_{s}+\frac{{V}_{b}}{t}$$

where *V*_*b*_, *V*_*s*_, and *V* are the bulk, surface, and total anomalous Nernst contributions, respectively and *t* denotes the thickness of the FM. Linear fit yields *V*_*s*_ = 5.47 *μV* and *V*_*b*_ = −29.51 *μVnm*. Hence bulk ANE with a 5 *nm* thick layer of Ni_7_Fe_93_ is about −5.90 *μV* and with a surface ANE contribution of 5.47 *μV*, results in a total anomalous Nernst signal of −0.43 *μV*, consistent with the data presented in Fig. 2(b). Therefore in the pursuit of a zero Nernst material, what we have truly achieved is the pivotal point where bulk ANE cancels off the surface ANE of the composition Ni_7_Fe_93_ at a thickness of 5 *nm*.

**Figure 5:**
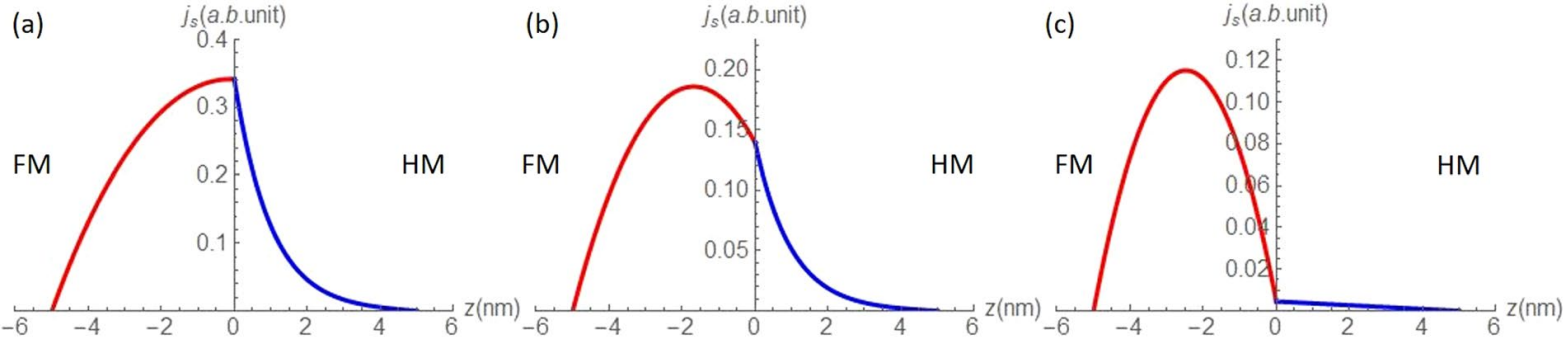
(**a**) Calculated spin current distribution for a FM/HM (Pt) bilayer in longitudinal configuration, where ${\lambda }_{HM}=1\,nm$, ${\lambda }_{FM}=5\,nm$, ${d}_{HM}=5\,nm$, ${d}_{FM}=5\,nm$, ${\sigma }_{HM}=1\,a.u.$, ${\sigma }_{FM}=1\,a.u.$, and $({S}_{\uparrow }-{S}_{\downarrow })\nabla T=1\,a.u.$ (**b**) Calculated spin current distribution for a FM/HM (Ta) bilayer in longitudinal configuration, where ${\lambda }_{HM}=1\,nm$, ${\lambda }_{FM}=5\,nm$, ${d}_{HM}=5\,nm$, ${d}_{FM}=5\,nm$, ${\sigma }_{HM}=0.1\,a.u.$, ${\sigma }_{FM}=1\,a.u.$, and $({S}_{\uparrow }-{S}_{\downarrow })\nabla T=1\,a.u.$ (**c**) Calculated spin current distribution for a FM/LM (Cu) bilayer in longitudinal configuration, where ${\lambda }_{HM}=50\,nm$, ${\lambda }_{FM}=5\,nm$, ${d}_{HM}=5\,nm$, ${d}_{FM}=5\,nm$, ${\sigma }_{HM}=1\,a.u.$, ${\sigma }_{FM}=1\,a.u.$, and $({S}_{\uparrow }-{S}_{\downarrow })\nabla T=1\,a.u.$

Many noteworthy work has been done to isolate SSE from a thermal measurement performed in the longitudinal configuration for a FM metal. Ramos *et al*.^27^ performed the thermal measurements on conducting films of magnetite, where they measured the ANE of magnetite directly and in the LSSE configuration. Further, estimated the ANE contribution due to proximity effect by dividing the Pt layer into magnetic and non-magnetic regions. Since magnetite in the metallic phase has a resistivity two orders of magnitude larger than that of Pt, ANE contribution of the magnetite is greatly suppressed by the Pt. In fact, authors report ANE contribution from the magnetite to the measured thermal signal is only 3%. This result cannot be extended to isolate the SSE from ANE in the present work, since FM and HM used here have resistivities of the same order. Jiang *et al*.^28^ studied the Topological Insulator (TI)/Yttrium Iron Garnet (YIG) structure. Where they eliminated the contribution from ANE to the measured thermal signal by choosing YIG, a ferrimagnetic insulator as the source of SSE. Methodology to remove proximity induced ANE signal from the TI here is very innovative. By means of anomalous Hall measurements, authors establish a critical temperature beyond which proximity induced anomalous Nernst contribution from TI layer is negligibly small, and performed SSE measurements at a temperature well beyond the critical temperature. However, TI/YIG system is starkly different from ours. Firstly, we use a FM metal as oppose to YIG, a ferrimagnetic insulator, where anomalous Nernst signal from the magnetic material cannot be avoided. Moreover, critical temperature to avoid proximity induced anomalous Nernst contribution from the TI is well below 300 K. Without extensive studies, we cannot guarantee such a methodology is suitable for an all metallic system. Even if such technique is suitable, we should pay attention to the appropriate critical temperature, as if this might be too high for a FM metal where it would lose its magnetic properties resulting in no signal. Xu *et al*.^29^ performed similar experiments with NiFe/Pd structure. In addition, they replaced the HM with β-Ta which is known to have a spin Hall angle opposite to that of Pd^30^, and yet observed a thermal signal with the same sign as Pd. Having eliminated proximity ANE by a Cu insertion layer, Xu *et al*. concluded, majority of the signal arose from the ISHE in the magnetic layer, which was caused by spin current redistribution in the magnetic layer induced by the presence of the probe layer. From Eq. (2) it is clear, that the neighboring heavy metal will significantly modify the spin current distribution in the FM. Figure 5 shows representative SSE driven spin current distributions calculated from Eq. (2). Though this result is appreciable, SSE has been separated from ANE only exploring the large thickness (*t* > 2*λ*) limit, detailed studies of the thin end is necessary to understand the full picture. With experiments performed probing the entire thickness range, it is clear, that the ANE of the isolated FM is not a pure bulk effect, rather contains a surface component, which will be modified by the presence of any neighboring layer. More importantly what is considered by Xu *et al*. as intrinsic ANE, coming from the bulk of the FM is not truly intrinsic rather changes with the introduction of any adjacent metal, particularly with an introduction of a HM.

Comparing data presented in Figs 2(b) and 3(a), we can observe some peculiar behavior. Polarity of the ANE signal measured for Ni_7_Fe_93_(5)/Cu(1) is opposite to that of Ni_7_Fe_93_(5). As we discussed earlier, Cu will not give rise to any SSE signal or any appreciable inverse spin Hall signal. Hence polarity change in the ANE signal, as a Cu layer is introduced on top the FM has to originate elsewhere. It is apparent from the analysis of the inverse thickness dependence of Ni_7_Fe_93_ shown in Fig. 4(b), ANE signal bears the sign of the dominant contribution of surface or bulk. Since at a thickness of 5 nm magnitude of the bulk contribution is larger than that of the surface, ANE signal carries the sign of the bulk contribution (negative). However, as a Cu layer is introduced on top of the Ni_7_Fe_93_(5), surface contribution is enhanced and if the magnitude of the enhanced surface contribution is larger than that of the bulk, ANE signal will now carry the sign of the surface contribution (positive). This is a strong evidence, reiterating the importance of the surface contribution to the ANE. Cu, layer thickness required to guarantee total surface coverage of the FM is about 2 nm. Cu placed on top of the FM will influence the surface until full surface coverage is reached and any Cu placed beyond this thickness does not influence the surface, rather only contribute to shunting. Therefore, surface contribution to the measured thermal voltage will vary until full surface coverage is reached and plateau thereafter, thus explaining the bump around 2 nm in the thermal voltage for Ni_7_Fe_93_(5)/Cu(x) shown in Fig. 3(b). Incidentally, spin diffusion length of Pt (*λ*_*Pt*_) is around 1.3 *nm* and, as shown in Fig. 3(b), measured thermal voltage for FM/Pt structure peaks around that thickness, hence appearing to have a peak position similar to that of FM/Cu.

## Conclusion

In summary, we employed the zero Nernst material approach to mitigate the ANE contribution in the thermal measurement of FM metal, and a thin Cu insertion layer to mitigate the magnetic proximity effect. These modifications were substantial in the process of developing a technique to isolate SSE from other parasitic effects in a FM metal. Our studies have confirmed that FM alloys can indeed have zero Nernst coefficient, which arises not only from the cancellation from, constituent elements having opposite Nernst coefficients but also from the competition among bulk and surface contributions. Effectively, zero Nernst coefficient is achieved when the bulk and surface contributions are equal but opposite in sign. The bulk contribution, which should be related with spin current distribution, will be modified with a good spin sink neighboring layer such as a HM layer. The surface contribution will also be affected by the neighboring layer arising from multiple factors such as spin accumulation, proximity effect, or density of states modification. Considering the magnitude of both bulk and the surface contributions in the isolated FM are approximately as large as 6 *μV*, which is comparable to the observed “SSE” signal around 8 *μV*, surface contribution to the ANE is a substantial effect in the thermal measurement of a FM metal. Our efforts uncovered the presence of both surface and bulk ANE and any future work aimed at isolating SSE from the thermal measurement of FM metals should take this phenomenon into consideration.
